# Revisiting soil bacterial counting methods: Optimal soil storage and pretreatment methods and comparison of culture-dependent and -independent methods

**DOI:** 10.1371/journal.pone.0246142

**Published:** 2021-02-10

**Authors:** Jeonggil Lee, Han-Suk Kim, Ho Young Jo, Man Jae Kwon

**Affiliations:** 1 KU-KIST Green School, Korea University, Seoul, Republic of Korea; 2 Department of Earth and Environmental Sciences, Korea University, Seoul, Republic of Korea; University College London Institute of Child Health, UNITED KINGDOM

## Abstract

Although a number of different methods have been used to quantify soil bacteria, identifying the optimal method(s) for soil bacterial abundance is still in question. No single method exists for undertaking an absolute microbial count using culture-dependent methods (CDMs) or even culture-independent methods (CIMs). This study investigated soil storage and pretreatment methods for optimal bacterial counts. Appropriate storage temperature (4°C) and optimal pretreatment methods (sonication time for 3 min and centrifugation at 1400 *g*) were necessary to preserve bacterial cell viability and eliminate interference from soil particles. To better estimate soil bacterial numbers under various cellular state and respiration, this study also evaluated three CDMs (i.e., colony forming unit, spotting, and most probable number (MPN) and three CIMs (i.e., flow cytometry (FCM), epifluorescence microscopy (EM) count, and DNA quantitation). Each counting method was tested using 72 soil samples collected from a local arable farm site at three different depths (i.e., 10–20, 90–100, and 180–190 cm). Among all CDMs, MPN was found to be rapid, simple, and reliable. However, the number of bacteria quantified by MPN was 1–2 orders lower than that quantified by CIMs, likely due to the inability of MPN to count anaerobic bacteria. The DNA quantitation method appeared to overestimate soil bacterial numbers, which may be attributed to DNA from dead bacteria and free DNA in the soil matrix. FCM was found to be ineffective in counting soil bacteria as it was difficult to separate the bacterial cells from the soil particles. Dyes used in FCM stained the bacterial DNA and clay particles. The EM count was deemed a highly effective method as it provided information on soil mineral particles, live bacteria, and dead bacteria; however, it was a time-consuming and labor-intensive process. Combining both types of methods was considered the best approach to acquire better information on the characteristics of indigenous soil microorganisms (aerobic versus anaerobic, live versus dead).

## Introduction

Soil is one of the most complex natural systems on the planet being a composite of various organic and inorganic constituents, including minerals, gas, water, and organic matter. These constituents provide the essential resources to promote the growth of a diverse range of soil bacteria. Because soil bacteria are highly abundant and diverse, they play an important role in shaping subsurface environments by controlling the primary metabolism and biogeochemical element cycles in these ecosystems [1]. Soil bacteria can grow and adapt rapidly to changes in the physicochemical properties of soil [2, 3]. Thus, soil bacterial abundance is closely related to properties such as pH, temperature, water, oxygen, organic matter content, and toxic compounds [4]. Bacterial abundance is also strongly dependent on soil heterogeneity, and is thus extremely site-specific in every soil environment even at the micro-scale. Quantification of soil bacterial abundance is essential for better understanding bacterial population dynamics and dominant soil biogeochemical processes.

Culturing methods have been traditionally used to estimate soil bacterial numbers [5, 6]. These culture-dependent methods (CDMs) measure the number of bacteria based on bacterial cultivability on various types of media. Compared to culture-independent methods (CIMs), these methods offer good accessibility to tools and are relatively inexpensive and easy to conduct. However, cultivable soil bacteria in a specific medium are considered to represent only 1.4–14.1% of the total average cell count based on microscopically determined methods [7]. For instance, CDMs are limited in being able to cultivate unculturable bacteria, such as viable but nonculturable (VBNC) microorganisms, and concurrently estimate aerobic and anaerobic bacteria. Only a small proportion of soil or rhizosphere microorganisms is considered to be cultivable, despite the use of various media [7, 8].

To overcome these limitations, several biochemical and molecular microbiological techniques including phospholipid fatty acid analysis (PLFA) [9], fluorescence *in situ* hybridization (FISH) [10], catalyzed reporter deposition-FISH [11], quantitative polymerase chain reaction (qPCR) [12], have been used to estimate soil microbial abundance. Epifluorescence microscopy (EM) [13] and flow cytometry (FCM) [14] have also been used as CIMs to count soil bacterial numbers by staining nucleic acids with fluorescent dyes. As CIMs do not require the cultivation of bacteria, bacterial abundance can be obtained quickly without waiting for cultivation periods. CIMs can also provide more accurate results as bacterial abundance is estimated using advanced analytical instruments (i.e., lower variation among replicates because of avoiding human error in detecting cell numbers). However, there may be limited accessibility to these methods due to the high costs of instrumentation and the associated reagents.

Prior to selecting an optimal method (considering the time saved, cost-effectiveness, difficulty, low variability between replicate samples, and information provided) to estimate bacterial abundance, it is important to consider the storage and pretreatment methods of soil samples to determine representative soil bacterial numbers. The storage temperature and storage time of the soil samples should not affect the soil bacterial numbers as the soil sample storage for an extended period of time at the temperature different from the soil’s *in situ* temperature may over- or under-estimate bacterial abundance. Additionally, pretreatment methods to separate bacteria from the soil matrix must be tested, as up to 70% of soil bacteria associate with soil microaggregates [15]. Bacterial numbers in aqueous solutions are relatively easy to measure due to minimal heterogeneity in the aqueous samples and zero interference from solid particles. In contrast, bacterial counts in soil samples are considerably difficult because of the extremely large extent of soil physicochemical heterogeneity as a result of potential micro-scale differences in various attributes, including soil water content, organic matter, and mineral particles. Therefore, it is necessary to develop reliable and optimal methods to preserve and pretreat heterogeneous soil samples to effectively estimate soil bacterial abundance.

The size of the soil sample number is another critical factor that influences the determination of representative bacterial abundance in specific soil sites. Although previous studies have attempted to compare various counting methods for soil bacteria, they only investigated a limited number of samples For example, only 5, 5, and 20 soil samples were tested for bacterial abundance in the studies by [16–18], respectively. Small sample sizes may leave large data gaps that may not effectively be able to link soil bacterial abundance to physical and chemical properties and identify the role of bacteria in soil.

In the current study, three CDMs (i.e., colony forming units (CFU), spotting, and most probable number (MPN)) and three CIMs (i.e., FCM, EM, and DNA quantification) were tested using 72 soil samples collected from Korea University research farm site. This large number of samples may provide higher density spatial coverage to reasonably ensure adequate representation of soil bacterial abundance at the site. This study also investigated optimal soil storage and pretreatment methods for better storage of soil samples and effective means of detaching bacteria from soil surfaces, respectively. The objectives of this study are to 1) identify the optimal soil storage temperature and pretreatment conditions for soil bacterial counts, 2) determine which CDMs or CIMs are more reliable and representative for the number of specific soil bacterial groups (i.e., aerobes, anaerobes, live cells, dead cells), and 3) evaluate whether bacterial abundance may be an indicator for soil microbial activities and processes (i.e., aerobic versus anaerobic bacterial abundance, and number of live versus dead bacteria).

## Materials and methods

### Sampling location

**Fig 1:**
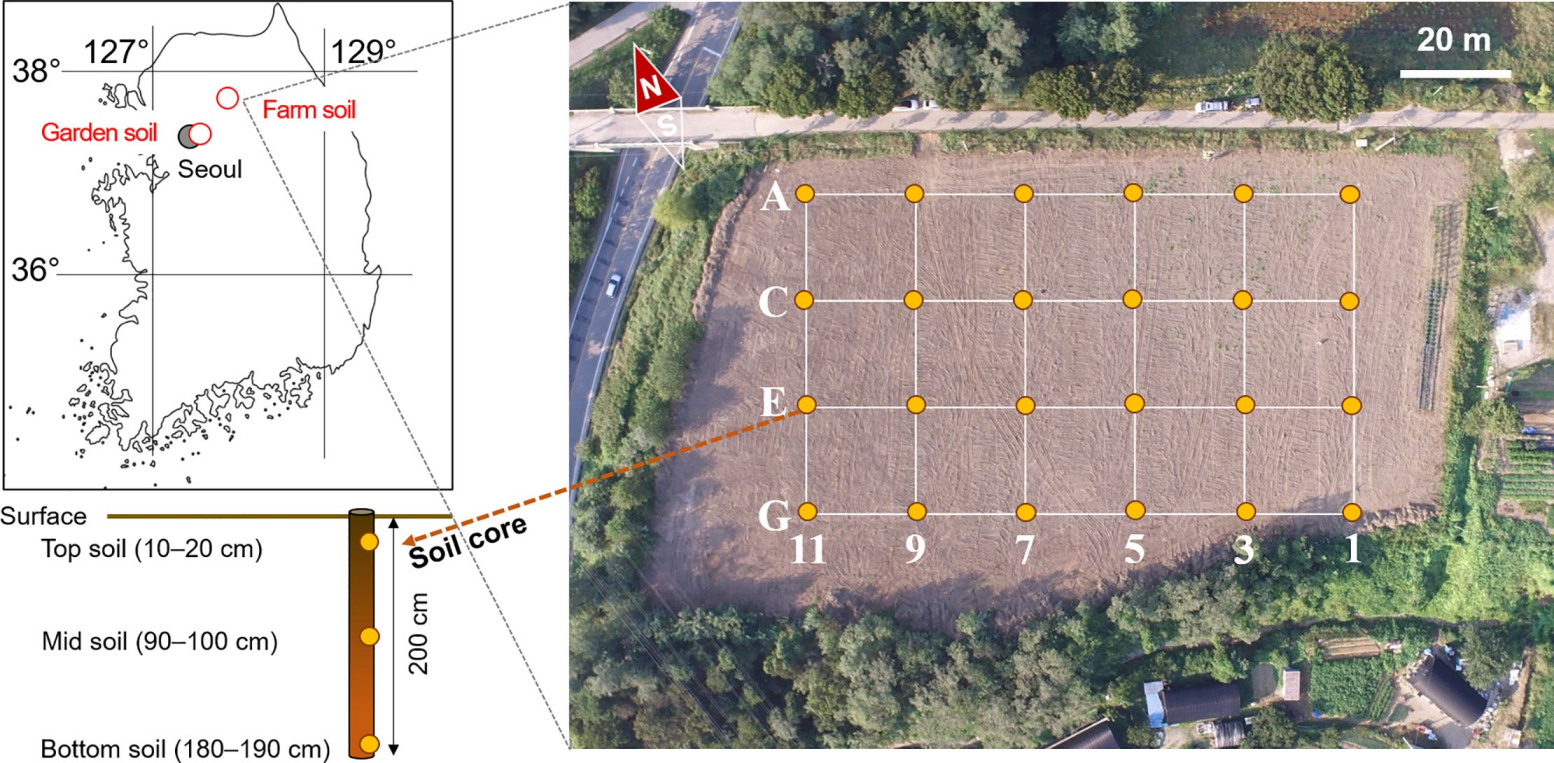
Study area and sampling points (garden and farm soil) for soil bacterial counts.

One set of soil samples were collected from a test-bed site (100 m length × 60 m width × 2 m depth) at the Deok-so research farm, Namyangju-si, Gyeonggi-do, Republic of Korea (Fig 1). The test-bed site was constructed to develop field strategies and technologies for integrated site characterization and managed by the SMART-SEM research center at the Korea University [19]. Since 1960, the site has been used to grow crops for cattle, using their fecal matter as fertilizer. A total of 24 core samples of 2 m length were collected from the site at 20 m interval along the line transects, using an undistributed core sampling machine (Fig 1). The soil cores were immediately transported to the laboratory. The subsamples were collected at the depths of 10–20, 90–100, and 180–190 cm using a knife sterilized with 70% ethanol. Approximately 15 g of each subsample were mixed thoroughly and transferred into sterilized 15 mL conical tubes after the removal of large gravel and roots to avoid the effects of bacteria living on roots [20]. Soil subsamples for CDMs and CIMs were stored at 4°C until analysis and samples to be processed via DNA quantification were stored at –20°C until DNA extraction.

**Fig 2:**
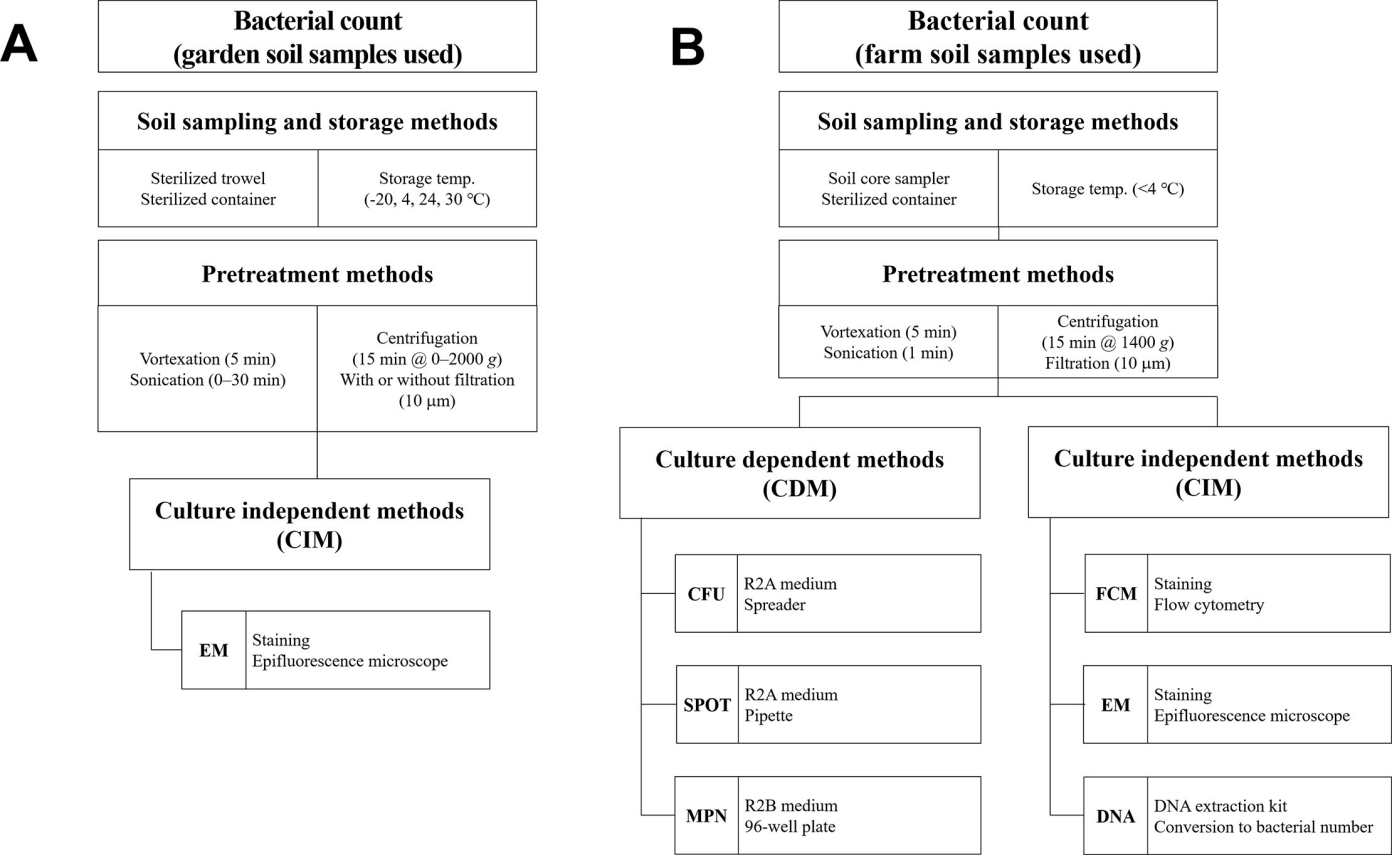
Experimental steps and methods for bacterial counts from garden (A) and farmland (B) soil samples.

To determine optimal pretreatment methods (such as effects of storage, vortexation, centrifugation, and filtration) for soil bacterial count, another set of soil samples were collected from a garden site at Korea University, Seoul, Republic of Korea (Fig 1) to reduce the time required to transfer and store the sample. Samples were collected using a sterilized trowel following removal of the top 5 cm of the soil layer, and were transferred into sterilized conical tubes. These samples were not refrigerated but were tested on the day of collection. Fig 2 and S1 Fig summarize the various pretreatment and counting methods for bacterial abundance in garden and farmland soil samples.

### Pretreatment

To separate the soil bacterial cells from the clay particles and organic matter in the soil matrix, 1 g of the soil sample was mixed with 9 mL of 0.22 μm filtered 0.85% saline (NaCl) solution in a 15 mL conical tube. The soil suspension was vortexed at maximum speed (~3000 rpm) for 5 min. Additional pretreatment tests were performed to determine the optimal pretreatment process using the garden soil samples (Fig 2A). The same pretreatment conditions as mentioned above were applied. The optimal sonication time for the microbial count was tested at 0, 1, 3, 5, 8, 10, 13, 15, 20, 25 and 30 min (3 × 20 s with 20 s intervals between pulses), with an ultrasonic bath (AC220 V/60 Hz, 300 W; Sae-Han ultrasonic, Korea). Centrifugation speed was also tested at 0, 100, 200, 400, 600, 800, 1000, 1200, 1400, 1600, 1800, 2000 × *g*. To determine the effect of filtration on bacterial count, soil samples were treated with and without filtration using a 10 μm filter paper (Whatman, UK). Bacterial numbers were counted by EM; the detailed EM method is described in Section ‘Staining and culture-independent methods’ below. All the numbers are expressed as cells per g soil dry weight hereafter.

Comparison of several bacterial counting methods were performed using soil samples from the farm site. The vortexed soil suspensions were sonicated for 3 min and centrifuged at 1400 × *g* for 15 min. The resulting bacterial cell suspension was then filtered using 10 μm filters (Fig 2B).

### Comparison of the effects of soil storage temperature

To test the effects of soil storage temperature, the garden soil samples (~100 g) were collected using sterilized 50 mL conical tubes and shaken by hand in a plastic bag to homogenize the samples as much as possible. The well-mixed soil samples were divided equally into four parts (20 g each), stored at –20, 4, 24, and 30°C, respectively. Equal amounts of subsamples (1 g) from the soil samples stored at different temperatures were transferred to 15 mL conical tubes using a sterilized spatula and further treated according to the pretreatment steps as described in the previous section (i.e., vortexation, sonication, centrifugation, and filtration). The stained bacterial numbers were measured by EM. The method of staining was mentioned in Section ‘Staining and culture-independent methods’ below.

### Culture media and condition

Soil bacteria were cultured in R2A culture media (Reasoner’s 2A agar, BD Difco™, US) for the CFU and spotting methods or R2A broth (R2B) media (R2A Broth is similar to R2A agar, except without agar) for MPN to measure general soil bacteria [21]. One liter of R2B culture media contained 0.5 g of yeast extract, 0.5 g of proteose peptone, 0.5 g of casamino acid, 0.5 g of dextrose, 0.5 g of soluble starch, 0.3 g of sodium pyruvate, 0.3 g of dipotassium phosphate, and 0.05 g of magnesium sulfate. 0.85% NaCl filtered through a 0.22 μm filter (Minisart Syringe Filter, Sartorious, German) was used as diluent solution. All cultivations were conducted under aerobic condition at 30°C for a week without shaking. All media were autoclaved at 121°C for 15 min before use.

### Culture-dependent methods

Pretreated samples (i.e., soil samples from the farm site treated with vortexation, sonication, centrifugation, and filtration) were diluted 3–5 times to obtain an appropriate cell number for each CDM. The last three diluted samples (~100 μL) were inoculated and spread evenly on R2A plates using a sterilized spreader until the medium was dry for CFU. After culturing the plates by reversing the petri dish (at 30°C for a week), bacterial numbers were counted when the number of colonies ranged between 30 and 300. As per CFU, spotting was performed after serial dilution of the original soil solution. Only 10 μL of each diluted sample was carefully dispensed on R2A plates and was left undisturbed. The dispensed droplets on the R2A plates were dried for approximately 5 min and incubated in an inverted position at 30°C for a week. Bacterial abundance was determined using the MPN method as described by [22]. Briefly, a 20 μL aliquot of the pretreated solution was added to the first row of a 96-well microtiter plate and serially diluted using a 1:10 dilution ratio to 2^nd^–8^th^ rows of the 96-well plate with 180 μL of R2B. The 96-well plate was incubated at 30°C for a week. A positive result for the MPN procedure was indicated by the development of turbidity in the well. All experiments for CDMs were performed in triplicate. A total of 648 agar plates for CFU, 72 agar plates for spotting, and 72 well plates for MPNs were used in these experiments.

### Staining and culture-independent methods

The resulting pretreated soil solution (~1 g dry wt. soil in 9 mL of 0.85% NaCl solution) with appropriate dilution (10^2^–10^4^) was stained with LIVE/DEAD^®^ BacLight^TM^ Bacterial Viability Kit (ThermoFisher SCIENTIFIC, USA) to be used for EM and FCM analysis. The pretreated soil solutions were stained with 3 μL mL^-1^ mixture of SYTO9 and Propidium iodide (PI) (3.34 mM of SYTO9:20 mM of PI = 1:1) and incubated for 15 min in the dark at room temperature. The FCM analyses were conducted on the BD FACS Accuri C6 plus (FL1 = 533/30 nm, FL2 = 585/40 nm, speed = slow 14 μL min^-1^, volume = 50 μL). To obtain accurate measurements, the bacterial concentration was maintained below 10^6^ cells mL^-1^ and 400 event s^-1^. To estimate the bacterial abundance using EM, 5 μL of a marked bacterial solution was placed on a slide glass. A cover slip was placed on the droplet and gently taped over it to eliminate air bubbles. Each scene was observed using green fluorescent protein and a RHOD filter (excitation 546 nm, emission 585 nm). Direct counts were made for at least 20 images at × 1000 with immersion oil. Past studies have demonstrated the conversion of the number of bacteria observed in EM to an estimate of bacterial abundance in soil (cell g^-1^) [23]. DNA was extracted by using a DNeasy® PowerSoil® Pro Kit (QIAGEN, Germany) according to the manufacturer’s directions, and the DNA concentration was quantified using a Qubit fluorometer (Invitrogen, USA). A conversion factor of 8.4 fg (10^−15^ g) DNA cell^-1^ was used to estimate the bacterial numbers based on the amount of DNA [24]. This estimation includes not only DNA from bacterial cells, but also extracellular or relic DNA in soil environments.

To investigate the effects of soil mineral particles on bacterial counts by FCM, additional experiments were conducted using pure cells, *Shewanella* sp. strain ANA-3. ANA-3 was aerobically cultured overnight on Luria-Bertani medium at 30°C with shaking. To make a sterilized sample, cultured ANA-3 was sterilized using an autoclave (121°C, 1.5 atm, 15 min). The cultured pure cell samples with or without sterilization were stained with LIVE/DEAD^®^ BacLight^TM^ Bacterial Viability Kit.

### Statistical analyses

Data are presented as the mean ± standard deviation. The Pearson correlation coefficient was calculated using SYSTAT 10.2 to determine the relationship between the estimated bacterial numbers and the method used (i.e., CFU, spotting, MPN, EM, and DNA quantification). Group differences were evaluated by one-way analysis of variance and T-test. In all analyses, *p* < 0.05 was the threshold for statistical significance.

## Results

### Effects of storage temperature and time on soil bacterial cell numbers

**Fig 3:**
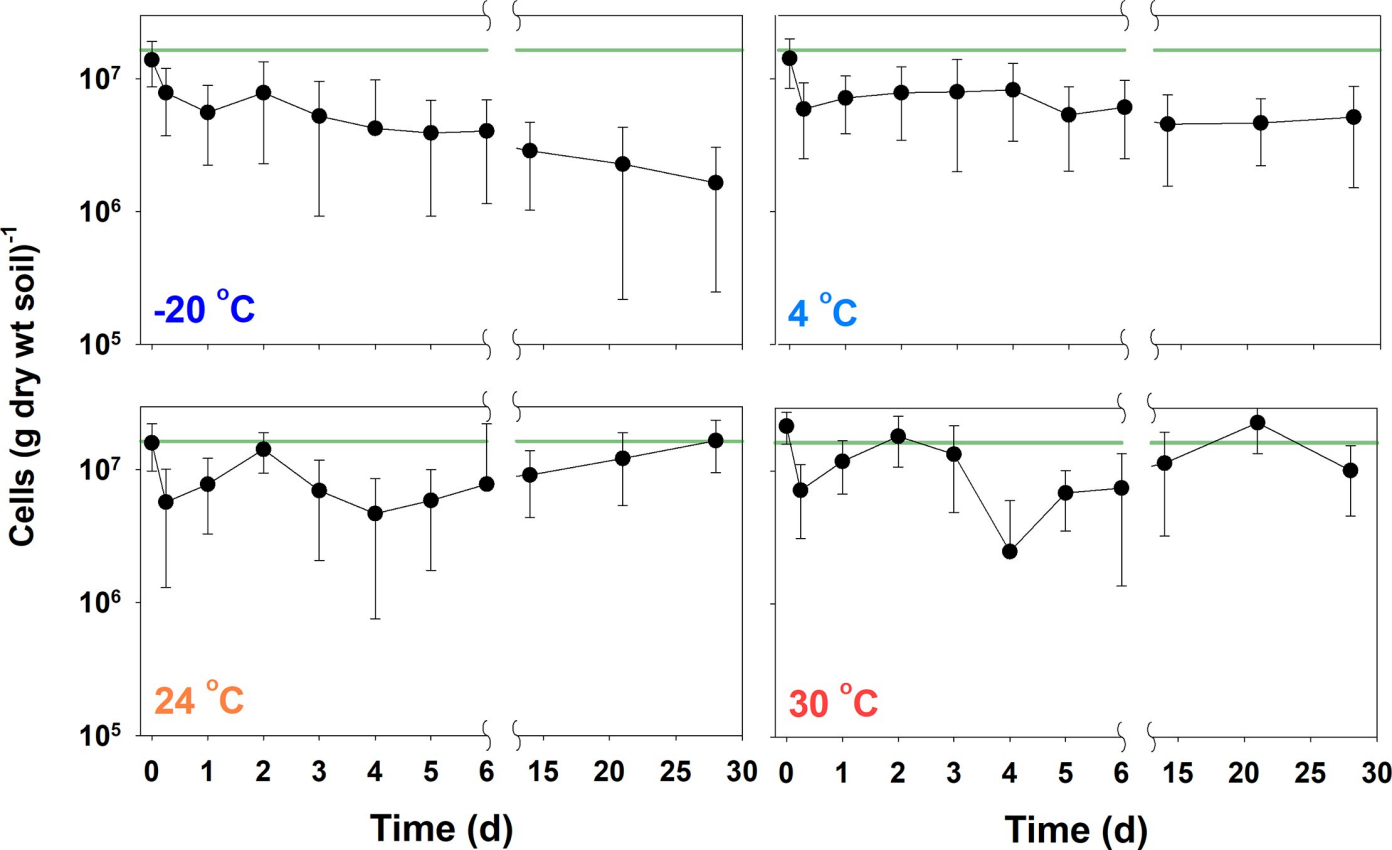
Effects of storage temperature on bacterial numbers. Results were obtained by epifluorescence microscopy (EM) using garden soil samples collected from Korea University. The green lines indicate the initial cell numbers in the soil sample counted within 1 h. All other pretreatment conditions were the same except the storage temperature; vortexed at maximum speed for 5 min, sonication at 300 W for 3 min, centrifugation at 1400 × *g* for 15 min, and filtration through 10 μm filters. Experiments were conducted in triplicate and data are shown as the mean ± standard deviation.

Soil storage temperature significantly impacted the bacterial numbers determined by EM at four different temperatures over time (Fig 3). A small variation was observed in the initial cell numbers (t < 1 h). Numbers decreased during the first 6 h of storage time at all tested temperatures. After an initial drop, bacterial numbers at –20°C continued to decrease over time and the numbers at 4°C remained relatively stable for four weeks. The cell numbers at storage temperatures of 24°C ± 1°C and 30°C fluctuated over the experimental period. In general, higher storage temperatures rendered a greater mean cell count.

### Effects of sonication time, centrifugation speed, and filtration on soil bacterial cell numbers

Sonication time decreased the bacterial abundance in the soil samples determined by EM (S2A Fig). The cell count was highest at a sonication time of 3 min, and 4-fold higher than without sonication. The cell count increased until the sonication time of 3 min, and then decreased. After 20 min of sonication, there was a large variation in the cell numbers. The cell number in the soil samples sonicated for 0.5 h was only 20% of the cell numbers without sonication.

As the centrifugation speed (× *gravity*) increased, the cell numbers decreased slightly over time (S2B Fig). However, the number of soil particles determined by EM was more than 10^8^ below 800 × *g* and the smallest number of soil particles was observed at 1400 × *g* (S3 Fig). As such, 1400 × *g* was selected as the optimal centrifugation speed.

Cell counts were similar regardless of filtration with the 10 μm filter (S2C Fig). Filtration through 10 μm filters was used for the rest of the experiments to remove any possible fine inorganic soil particles (e.g., clays).

### Effects of different culture-dependent methods on soil bacterial cell numbers

**Fig 4:**
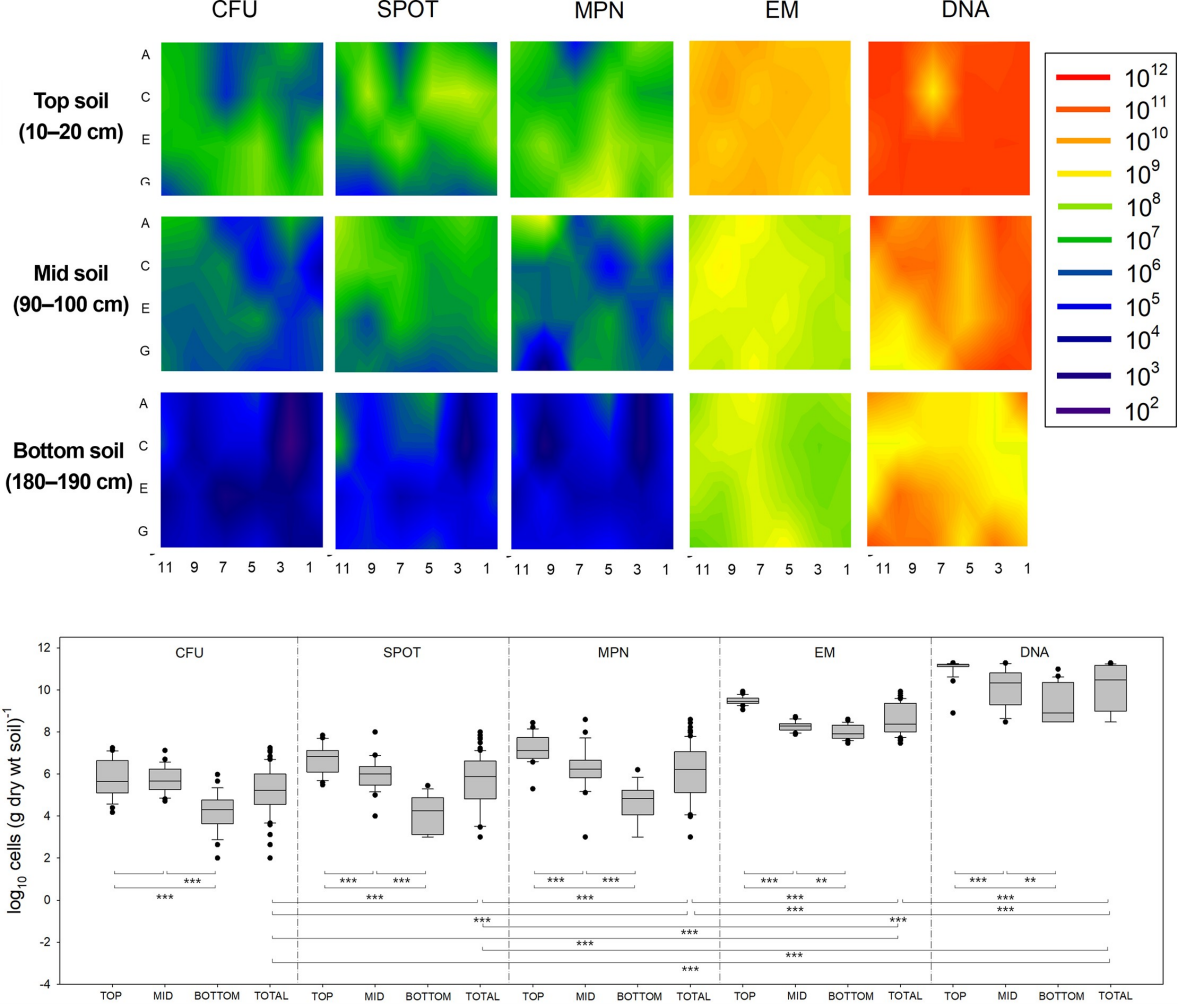
Contour and bar plots showing the distribution of bacterial numbers at three depths of farmland soil samples. The numbers were determined by five different methods (CFU, spotting, MPN, EM, DNA quantification). These soil samples were stored at 4°C and pretreated by vortexation at maximum speed for 5 min, sonication at 300 W for 3 min, centrifugation at 1400 × *g* for 15 min, and filtration through 10 μm filters. Experiments of bacterial counts were conducted in triplicate. *p < 0.05, **p < 0.01 and ***p < 0.001.

Cell counts using CFU had the lowest bacterial abundance of all CDMs tested in this study (Table 1 and Fig 4). Bacterial numbers obtained by the spotting were measured on considerably small parts of the plate (droplet) (S1 Fig). The results demonstrated that 30 samples (42%) of the 72 soil samples exhibited more than 50% variability (i.e., standard deviation), in comparison with the mean bacterial numbers (i.e., average in triplicate measurements for each soil sample). There was relatively large variability in the spotting method in comparison with the other CDMs (S4 Fig). MPN required the least amount of time among all CDMs and estimated 10^1^–10^3^ more bacterial abundance than other CDMs.

**Table 1:** Average of bacterial number in each soil depth with five different methods (CFU, spotting, MPN, EM, DNA quantification).

	Culture-dependent methods [cells (g dry wt soil)^-1^]			Culture-independent methods [cells (g dry wt soil)^-1^]	
	CFU^a^	Spotting	MPN^b^	EM^c^	DNA^d^ quantitation
**Top soil (10–20 cm) (n = 24)**	1.6×10^7^±2.3×10^7^	3.4×10^7^±5.7×10^7^	5.4×10^7^±8.0×10^7^	4.0×10^9^±1.9×10^9^	1.6×10^11^±5.7×10^10^
**Mid soil (90–100 cm) (n = 24)**	2.5×10^6^±3.2×10^6^	1.8×10^7^±3.4×10^7^	2.8×10^7^±9.5×10^7^	2.6×10^8^±1.4×10^8^	6.2×10^10^±8.0×10^10^
**Bottom soil (180–190 cm) (n = 24)**	9.0×10^4^±1.9×10^5^	1.1×10^6^±2.6×10^6^	2.3×10^5^±4.5×10^5^	4.6×10^8^±1.3×10^8^	1.6×10^10^±2.8×10^10^
**Total (n = 72)**	6.2×10^6^±1.5×10^7^	1.8×10^7^±4.0×10^7^	2.7×10^7^±6.2×10^7^	1.5×10^9^±2.1×10^9^	8.1×10^10^±8.5×10^10^

^a^ CFU: colony forming units.

^b^ MPN: most probable numbers.

^c^ EM: epifluorescence microscope.

^d^ DNA: deoxyribonucleic acids.

These soil samples were collected from the farm site and were stored at 4°C and pretreated by vortexation at maximum speed for 5 min, sonication at 300 W for 3 min, centrifugation at 1400 × *g* for 15 min, and filtration through 10 μm filters.

### Effects of different culture-independent methods on soil bacterial cell numbers

**Fig 5:**
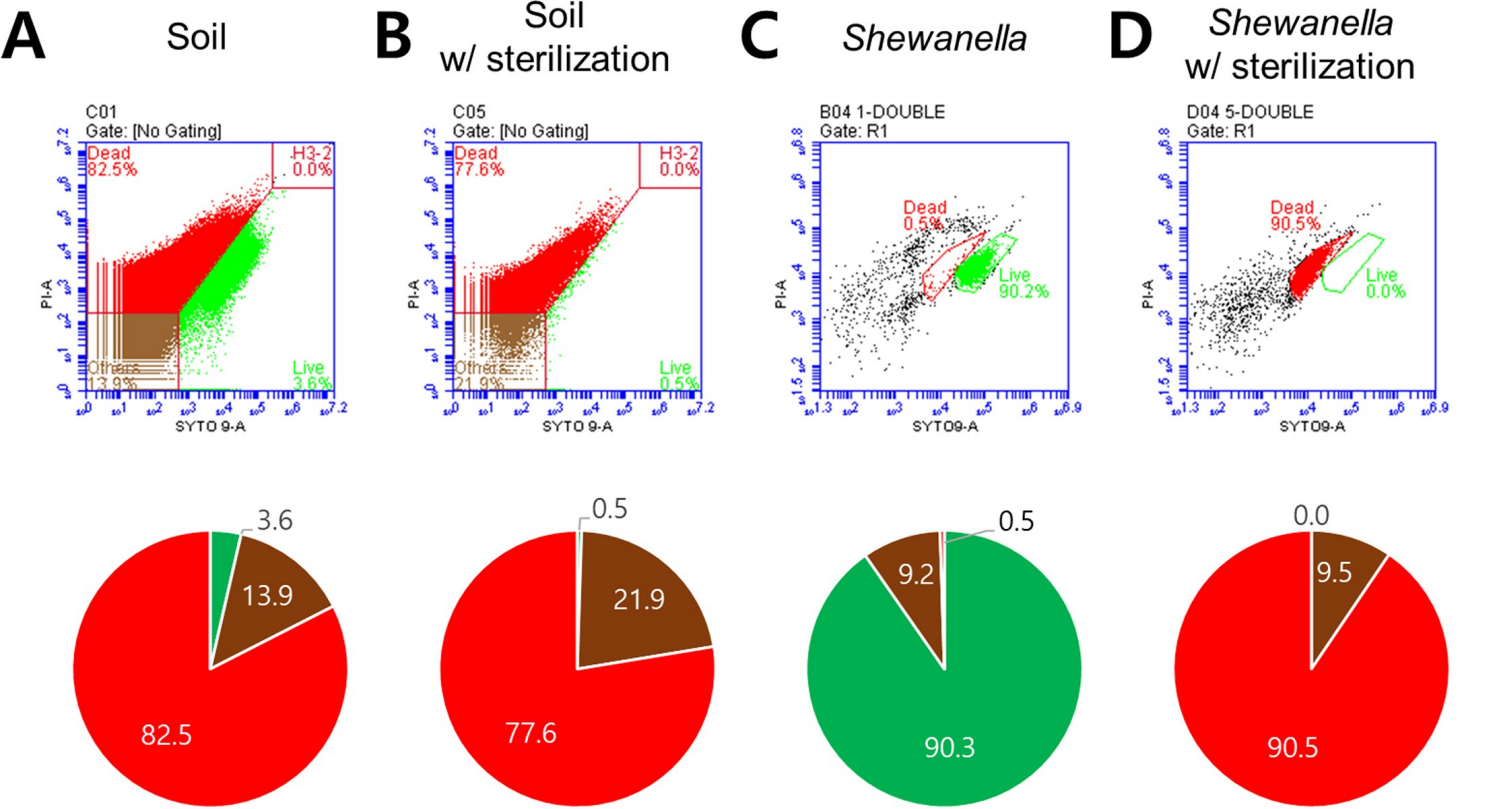
Scattered graphs (A and C) and pie charts (B and D) of bacterial numbers determined by FCM. The results of A and B indicate the bacterial numbers in the farmland soil sample (A1 at 10–20 cm), and those of C and D show the bacterial numbers in the single cell cultured media (*Shewanella* sp. in R2A media). Live cells, dead cells, and soil mineral and organic matter particles are shown in green, red, and brown, respectively. The samples were stored at 4°C and pretreated by vortexation at maximum speed for 5 min, sonication at 300 W for 3 min, centrifugation at 1400 × *g* for 15 min, and filtration through 10 μm filters.

The results of FCM indicated that soil bacteria were mostly dead (Fig 5). For the soil sample 1A at 10–20 cm, most particles presented in the soil extractant fluoresced red (82.5%), 3.6% fluoresced green and 13.9% did not fluoresce (Fig 5A). When the same soil sample was heat-sterilized (i.e., autoclaved at 121°C for 15 min), 77.6 and 0.5% fluoresced in red and green, respectively (Fig 5B). To investigate the effects of soil mineral particles (e.g., clays) on bacterial counts by FCM, cultivated pure cells (*Shewanella* sp. strain ANA-3) were used. Without sterilization, 90.3% of the total particles in the pure strain culture fluoresced green, 0.5% fluoresced red, and 9.2% did not fluoresce (Fig 5C). However, when *Shewanella* was heat-sterilized, 0.02% fluoresced green and 90.5% fluoresced red (Fig 5D).

**Fig 6:**
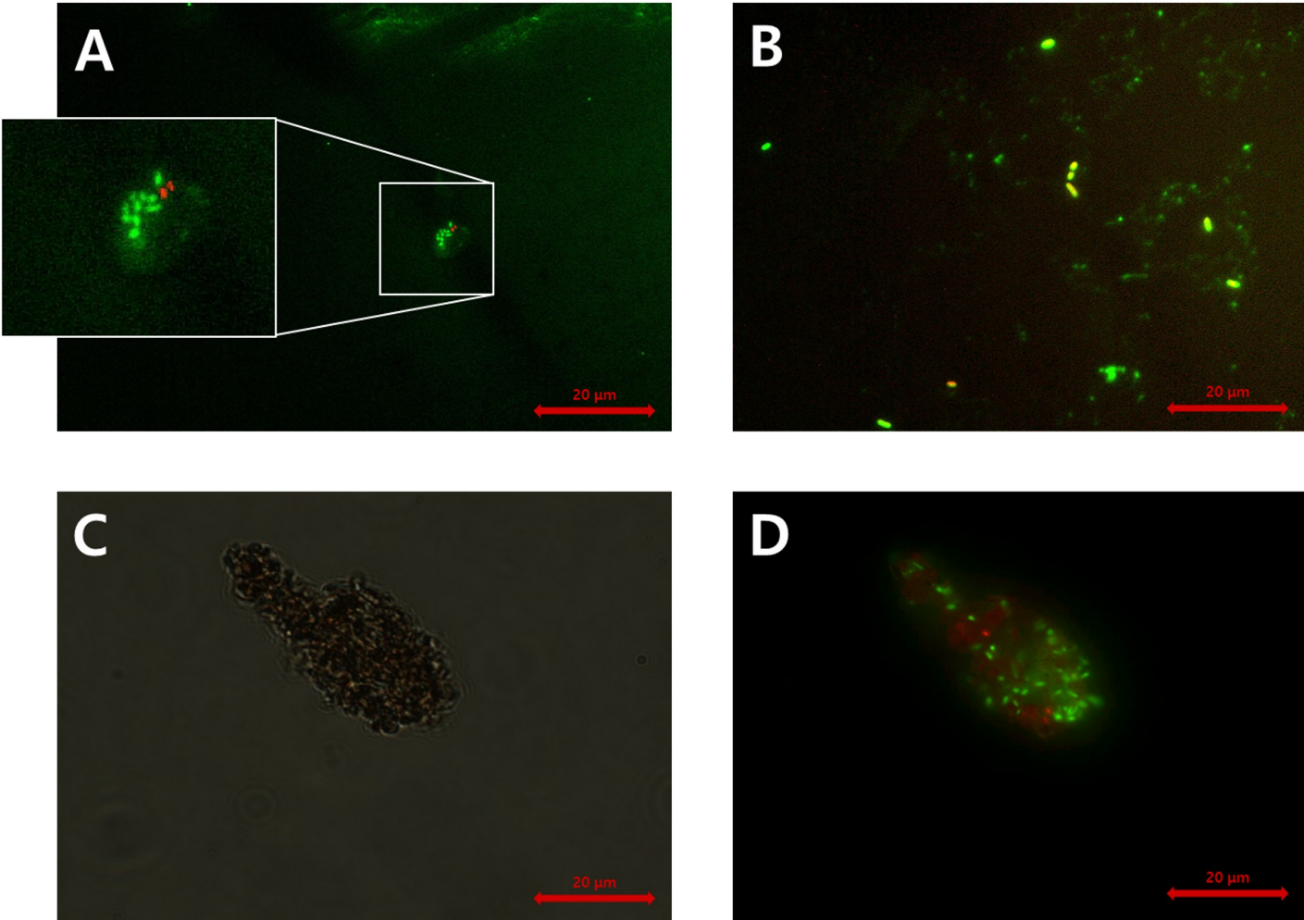
Photographs of bacteria and soil particles under EM. The photographs of A and B show bacterial cells in the garden soil samples filtered through 10 μm filters, and those of C and D show bacterial cells attached on the soil microaggregate in the soil samples without filtration. The photograph of C was taken under differential interference contrast (DIC) mode, whereas those of A, B, and D were taken under fluorescence mode. Live cells, dead cells, and damaged cells are shown in green, red, and yellow, respectively. The samples were stored at 4°C and pretreated by vortexation at maximum speed for 5 min, sonication at 300 W for 3 min, and centrifugation at 1400 × *g* for 15 min.

Stained soil extracts were also observed by EM (Fig 6). The results of EM indicated that some bacteria in the extractant emitted both green and red fluorescence (i.e., yellowish fluorescence; Fig 6B). In this study, bacteria with yellowish fluorescence were counted as live bacteria. A large number of bacteria was estimated with EM in comparison with FCM and the former could distinguish between live and dead bacteria and between soil particles and bacteria (i.e., soil particles were stained with SYTO9 or PI). However, the observational time for EM was approximately four-times greater than that for FCM (e.g., 0.8 h versus 0.18 h). The estimated bacterial abundance in the soil samples using the DNA quantification method was the highest amongst all CDMs and CIMs in this study (Fig 4). The DNA quantification method did not require the soil sample pretreatment steps as other methods. However, additional steps, including DNA extraction from soil, DNA purification, and DNA quantification, were required.

## Discussion

### Effect of storage temperature and time on soil bacterial numbers

In general, the cell count increased with soil storage temperature. The cell count at a higher temperature significantly varied over a month. This is unsurprising as faster cell growth can occur at higher temperatures and soil may have various types of cells with different growth rates and characteristics. At all temperatures, the numbers of live bacterial cells decreased within 6 h likely because of the change in temperature in soil samples from the original soil temperature (~18°C). These changes to the initial temperature condition, as well as changes to humidity and oxygen levels may have been a stressor for bacterial viability [25]. Cell numbers continued to decrease over a month when the soil samples were stored at –20°C, suggesting that freezing the soil samples over a long period of time leads to a significant underestimation of the *in situ* soil bacterial abundance. As the number of bacteria stored at 4°C exhibited the least variance for up to 4 weeks in comparison with other storage temperatures, soil storage at 4°C was suitable for the storage of soil samples used to estimate bacterial abundance. Nevertheless, it is always desirable to undertake this estimation as soon as possible, as certain physicochemical changes in soil samples can occur during the short or long-term storage of soil samples.

### Effect of soil pretreatment methods on soil bacterial numbers

Bacterial desorption from soil media and the elimination of soil particles and microaggregates prior to cell counting is essential to estimate the representative bacterial abundance. The association of bacteria with soil particles and microaggregates may interfere with CDMs and CIMs or cause difficulty in distinguishing between particles and cells, leading to an overestimate or underestimate of cell counts [26]. Currently, there is no agreement as to which procedure provides the best detachment of bacteria from the media [14, 27, 28] because of different soil characteristics. Cell numbers were 4-fold higher with 3 min of sonication than without sonication (S2A Fig). Beyond 3 min, the number of bacteria continued to decrease until 30 min. This suggests that prolonged exposure of bacteria through sonication kills or damages bacterial cells. These tendencies are similar to a previous study [29] in which the highest number of estimated bacteria were observed after 5 min of sonication. These results indicate that using an optimum sonication time is essential to accurately determine bacterial abundance. Bacterial cells prefer to attach to the soil particle surfaces, including natural organic matters and soil minerals; however, each soil type is different in terms of the amounts and composition of soil particles. Thus, prior to the determination of bacterial numbers, the optimal sonication time should be pre-determined as it may be site-specific.

Cell numbers were highest without centrifugation as expected, but decreased by 0.5% at 1400 × *g* (S2B Fig). A previous study also found that centrifugation speed up to 2000 × *g* had a negligible effect on bacterial enumeration in manure samples [30]. In our study, the number of other soil particles, such as soil minerals, was much higher with lower speed centrifugation, and was the lowest at 1400 × *g* (S3 Fig). Soil particle numbers were observed to be 10 times higher with lower speed centrifugation (0 to 600 × *g*). These results suggest that centrifugation is one of the critical pretreatment steps for bacterial cell counts in soil samples as soil mineral particles may interfere with bacterial cell counting, particularly by CIMs.

The number of bacterial cells was similar regardless of whether or not membrane filtration was used (S2C Fig). To remove large soil particles, a 10 μm pore size filter was used as most soil bacteria are less than 5 μm [31]. Supernatants of the centrifuged samples were expected to contain large amounts of microaggregates and soil particles. However, results suggested that membrane filtration through 10 μm filters was not critical to eliminate soil mineral particles after centrifugation. This applies only to specific soil samples, and as such, careful consideration is required to determine whether filtration is required.

### Differences in bacterial numbers among culture-dependent methods

There are several advantages of CDMs over CIMs. First, CDMs are less affected by the presence of soil particles such as clay and microaggregates [14] as bacteria can grow regardless of the presence or absence of soil particles. Second, CDMs are easy to conduct as they do not need specific instruments. However, the estimation of bacterial abundance using CDMs is influenced by the technical skills of the personnel undertaking the method. Additionally, CDMs are limited to counting bacterial cells that grow in specific media, potentially missing important unculturable bacteria [32].

Among the three CDMs, MPN exhibited the highest bacterial cell counts at all depths with the lowest variability (Table 1, Fig 4, and S4 Fig). This may be because MPN counts VBNC during the incubation of soil samples with liquid media. A previous study demonstrated that VBNC can be recovered with more than 3 days of incubation of soil bacteria using MPN [33]. MPN can also be combined with other molecular methods to better quantify subsurface environmental bacteria. Polymerase chain reaction (PCR) after MPN (MPN-PCR) was used to estimate soil bacterial number and characterize culturable community compositions [34]. Although the spotting method was relatively rapid as it does not require spreading, it exhibited the largest error among all CDMs (S4 Fig). This large error may be due to the overlap of colonies from many soil bacteria with different cell sizes and growth rates [35]. CFU requires the preparation of agar media and many plates and the spreading of bacterial solution using spreaders, which was found to be considerably labor-intensive and time-consuming. However, CFU exhibited better colony morphology, including colony size, shape, texture, elevation, and pigmentation, thereby aiding in the identification of bacterial species in general.

In summary, the optimal CDM can be selected based on the lowest variation of bacterial cell numbers among replicates. The MPN method showed the lowest variation of bacterial cell numbers, independent of over-growth or overlap of bacterial colonies, without the need to prepare agar plates. As such, MPN is recommended for the estimation of live bacterial numbers in soil samples.

### Differences in bacterial numbers among culture-independent methods

DNA quantification estimated the highest bacterial abundance among the six quantitative methods (Table 1 and Fig 4). A previous study estimated greater soil microbial richness up to 55% when using DNA recovered from soil, because of extracellular DNA (not from intact cells) in the soil samples [36]. These extracellular or relic DNA can be one of the largest pools of DNA in soil. Although these relic DNA can affect a number of important ecological and evolutionary processes in soil bacteria community, it may also affect measures of bacterial abundance [37]. This suggests that careful interpretation should be made when DNA quantification is used as the bacterial counting method. The higher number of bacterial cells determined by DNA quantitation is also likely due to greater resolution of the DNA-based technique in identifying bacterial genotypes than other methods. Therefore, it would be beneficial to compare the estimates by DNA quantification with those by other counting methods.

FCM was unable to clearly distinguish between live and dead bacteria and soil particles. Differentiation could be achieved based on the degree of staining by SYTO9 (live cell) and PI (dead cell); however, these dyes stained the live/dead bacteria and soil particles. The bacterial viability kit can distinguish live bacteria from dead or damaged bacteria as a function of bacterial membrane integrity. PI is impermeable to undamaged membranes (i.e., live cells); thus, it can only invade into cells with damaged membranes and bind to DNA. In contrast, green fluorescing SYTO9 can permeate all cell membranes, thus staining all bacterial DNA. When two stains are present in the bacterial DNA, SYTO 9 will be displaced from the nucleic acids and the bacteria will fluoresce in red because PI has more affinity to DNA than SYTO9. Thus, this kit can visualize and distinguish between the living and damaged bacteria. The FCM results indicated that soil samples fluoresced red (> 82.5%), likely representing dead bacteria (Fig 5A). However, observation with EM suggested that this red fluorescence was not only because of the dead bacteria, but also because of the soil particles stained by PI. FCM measured significantly lower live bacterial numbers than EM (S6 Fig). This may be because PI interferes with the SYTO9 fluorescence [38], microaggregates, and characteristics of an instrument that cannot measure less than a certain size. Thus, FCM may lead to an underestimate of live bacterial numbers (S6 Fig).

The EM observations at × 1000 and × 400 magnifications could distinguish between bacteria and soil particles (Fig 6). Although EM was considered as a tedious method (20–30 photographs should be taken per sample), it was considered the powerful method [39]. Our study also suggests that EM was the most appropriate for quantifying soil bacteria of all tested CIMs in this study.

### Comparison of soil bacterial numbers by CDMs and CIMs

CIMs generally have a more rapid turnaround of results than CDMs. CIMs also enable the concurrent estimation of anaerobic and aerobic bacteria. Contour plots of bacterial numbers measured by five different methods demonstrated that the number decreased with depth (Fig 4) similar to that reported in other studies [40, 41]. CIMs counted 10^3^–10^5^ times more bacteria than CDMs, and the estimated bacterial numbers were in the order of DNA > EM > MPN > spotting > CFU. If we assume that 82.5% of soil bacteria were all dead bacterial cells (Fig 5A), the dead cell numbers could account for a discrepancy of two to four orders of magnitude of the cell numbers between CDMs and CIMs. Differences in the bacterial numbers between CDMs and CIMs were more prominent at a deeper depth (i.e., 80–190 cm) than at shallower depths (i.e., 10–20 cm and 90–100 cm). This suggests that CIMs may count anaerobic bacteria as well as aerobic bacteria in the soil samples collected at a deeper depth. This result also supports that another order of magnitude discrepancy in the cell numbers between CDMs and CIMs is because of the difference between ‘live’ aerobic and anaerobic bacterial cell numbers. In addition, the data showed the higher variation of bacterial cell numbers determined by CDMs at deeper depths, suggesting that CDMs are better for counting aerobic bacteria rather than anaerobic bacteria (S4 Fig).

Three CDM methods demonstrated strong correlations in most cases (total Pearson correlation- CFU versus spotting = 0.66; CFU vs MPN = 0.91; spotting vs MPN = 0.55); however, two CIMs indicated weak correlations (EM versus DNA = 0.39; Table 2). Additionally, the correlations between CDM and CIM were weak to moderate (total Pearson correlation- CFU versus EM, DNA = 0.54, 0.45; spotting versus EM, DNA = 0.27, 0.30; MPN versus EM, DNA = 0.52, 0.50). This may be because all three CDMs count only culturable aerobes (on R2A or R2B), exhibiting similar patterns, whereas CIMs count all bacteria. In particular, the DNA quantification method can count extracellular DNA derived from all organisms in soil samples (e.g., dead wood and cattle rumen and manure) [42], which may be the cause for the difference in the distribution pattern between EM and DNA quantification and the overestimation of bacterial abundance.

**Table 2:** Summary of Pearson correlations (n = 72) between five different bacterial counting methods and soil properties.

Total (n = 72)	CFU^a^	Spotting	MPN^b^	EM^c^	DNA^d^	Top soil (n = 24)	CFU	Spotting	MPN	EM	DNA
CFU	1	0.66*	0.91*	0.54*	0.45*	CFU	1	0.26	0.83*	0.69*	0.70*
SPOT		1	0.55*	0.27*	0.30*	SPOT		1	0.15	0.19	0.24
MPN			1	0.52*	0.50*	MPN			1	0.73*	0.73*
EM				1	0.39*	EM				1	0.73*
DNA					1	DNA					1
Mid soil (n = 24)	CFU	Spotting	MPN	EM	DNA	Bottom soil (n = 24)	CFU	Spotting	MPN	EM	DNA
CFU	1	0.48*	0.72*	-0.05	0.05	CFU	1	0.31	0.88*	0.20	0.03
SPOT		1	0.36	-0.08	0.35	SPOT		1	0.24	0.14	-0.14
MPN			1	-0.26	0.11	MPN			1	0.10	0.23
EM				1	0.00	EM				1	–0.07
DNA					1	DNA					1

^a^ CFU: colony forming units.

^b^ MPN: most probable numbers.

^c^ EM: epifluorescence microscope.

^d^ DNA: deoxyribonucleic acids quantification.

(r = ±(0.5–0.7), moderate relation; r = more or less than ± 0.7, strong relation; *p < 0.05). These soil samples were collected from the farmland site and were stored at 4°C and pretreated by vortexation at maximum speed for 5 min, sonication at 300 W for 3 min, centrifugation at 1400 × *g* for 15 min, and filtration through 10 μm filters.

Cost and efficiency of each method were calculated and compared based on the analysis of 72 soil samples conducted in this study (S1 Table, estimated in September 2020). The CFU and spotting methods were characterized by a relatively easy experimental process and lower costs (CFU- $703 USD and SPOT- $9 USD); however, they may also be inaccurate and provide less information with the obtained results. CFU was the most time-consuming out of the tested CDMs and CIMs (CFU- 260 h required), whereas spotting was faster than CFU (180 h). MPN was relatively easy to perform, had a lower cost ($122 USD), and provided relatively consistent numbers and additional information. MPN also required less time than CFU and the spotting method (177 h). If only one sample was measured, the FCM method might be the most time-saving (0.08 h); however, the number of samples to be measured was extremely large (864 samples). It was also found to have a low accuracy and provided minimal information (i.e., could not differentiate between live bacteria, dead bacteria, and soil particles), and was the most expensive (total $19 544 USD) and difficult to perform. The DNA quantification method provided details on the number of bacteria and the bacterial communities present, was easy to perform, but was found to have a low accuracy in estimating bacterial numbers (i.e., it measured all the live and dead bacteria at once). The EM method provided detailed information (i.e., it could measure live bacteria, dead bacteria, and soil particles separately), exhibited high accuracy, and was time-saving and easy to conduct (39 h); however, it was relatively expensive in comparison with CDMs (total $2249 USD).

Each counting method exhibited several advantages and disadvantages. The results of our study suggest that bacterial numbers determined by both CDMs and CIMs provide useful information for better assessment of soil microorganisms, including aerobic and anaerobic bacteria and cellular states.

### Other soil bacterial quantification methods

In addition to the six methods investigated in this study, many other methods are available to estimate soil bacterial abundance. The PLFA method analyzes and estimates bacterial abundance in soil samples. Phospholipids are vital components of bacterial membranes and differ between eukaryotes and prokaryotes. Thus, this method provides an insight into the microbial community compositions as well as microbial numbers [9]. The FISH method is a molecular cytogenetic technique observing native microbial populations and using fluorescent probes [43] that bind to parts of the nucleic acid sequence on the root surface and the rhizoplane of paddy soil [44]. Bacterial fluorescence may be observed weakly by plant tissues and soil particles, but catalyzed reporter deposition (CARD)-FISH using catalyzed reflectors has been used to overcome this problem [45]. The qPCR method is used to quantify nucleic acids based on the real-time detection of a reporter molecule whose fluorescence increases as the PCR product accumulates during each amplification cycle. The qPCR method is a relatively rapid yet quantitative assessment of bacterial numbers for specific phylogenetic groups of microorganisms in soil [46]. However, the use of copy numbers of 16S rRNA genes in regard to bacterial cell counting remains uncertain because many bacteria contain different number (1 to 15) of the 16S rRNA genes, leading to incorrect estimation of bacterial cell numbers [47]. In addition, absolute quantification of the target genes using qPCR can result in biased outcomes caused by losses during DNA extraction or by co-extracted compound. To improve qPCR estimation, relative quantification by application of target to reference genes ratio (or of an artificial reference spike) was performed [48].

To assess bacterial abundance, these molecular methods and other bacterial counting methods have been individually investigated for many decades. Our study investigated and compared several conventional counting methods using CDMs and CIMs with many soil samples. Our study presents the advantages and disadvantages of each method and provides several important guidelines for selecting simple but reliable methods for estimating bacterial numbers in heterogeneous soil samples. This study also highlights that no single method is perfect for measuring soil bacterial cell numbers; the best counting method depends upon the question being asked and the lower variability among replicates.

## Conclusion

Appropriate storage temperature (4°C) and optimal pretreatment methods (i.e., sonication time, centrifugation speed, filtration) were necessary to preserve bacterial cell viability and eliminate interference from soil particles. With these optimal pretreatment methods, CDMs and CIMs were used to estimate bacterial cell numbers using a high-density of soil samples. CFU, MPN, and spotting were selected as CDMs, and EM, FCM, and DNA quantification were selected as CIMs. Methods were evaluated based on various criteria, including experimental time, cost, difficulty, available information, and data variability. Among CDMs, MPN was the most appropriate (i.e., easy, cheap and less labor-intensive), whereas EM was the most appropriate among CIMs (i.e., time efficient and more information provided). This study’s findings suggest that using both CDMs and CIMs can provide a better characterization of indigenous bacterial abundance in soil and their potential impacts on subsurface environments.

## Supporting information

S1 Fig Graphical summary of various pretreatment and counting methods for bacterial numbers in soil samples. (DOCX)

S2 Fig Effects of sonication time (a), centrifugation speed (b), and filtration (c) on soil bacterial number during the pretreatment processes. Pretreatment effects were tested by epifluorescence microscope using the garden soil samples collected from around Korea University. These soil samples were stored at 4°C. The pretreatment conditions for sonification time effects (a) included vortexing at maximum speed for 5 min, centrifugation at 1400 × *g* for 15 min, and filtration through 10 μm filters. The pretreatment conditions for centrifugation speed effects (b) included vortexing at maximum speed for 5 min, sonication at 300 W for 3 min, and no filtration. The pretreatment conditions for filtration effects (c) included vortexing at maximum speed for 5 min, sonication at 300 W for 3 min, centrifugation at 1400 × *g* for 15 min, with or without filtration through 10 μm filters. Experiments were conducted in triplicate.
(DOCX)

S3 Fig Effects of centrifugation speed on soil bacterial number during the pretreatment processes. Bacterial cells were counted by epifluorescence microscope using the garden soil samples collected from around Korea University. These soil samples were stored at 4°C. The pretreatment conditions for centrifugation speed effects included vortexing at maximum speed for 5 min, sonication at 300W for 3 min, and no filtration. Experiments were conducted in triplicate.
(DOCX)

S4 Fig Variability rates (%) of microbial cell numbers determined by MPN, spotting and CFU at each soil depth in the farmland. The rates were calculated by [(Standard deviation /Average)×100]. Experiments were conducted in triplicate.
(DOCX)

S5 Fig Scatter graphs were obtained by FCM with three farmland soil samples. Each row displayed a different soil. A) No staining; B) SYTO9 staining; C) PI staining; and D) both SYTO9 and PI staining. Experiments were conducted in single.
(DOCX)

S6 Fig Comparison of live bacterial cell numbers in farmland soil samples determined by MPN, EM, and FCM. The samples were stored at 4°C and pretreated by vortexing at maxim speed for 5 min, sonication at 300 W for 3 min, and centrifugation at 1400 × *g* for 15 min. Experiments were conducted in triplicate.
(DOCX)

S1 Table Summary of cost, experimental time, difficulty, and labor intensiveness of each method and potential information obtained from each method. Comparison of each method was based on a total of 72 soil samples from the farmland. Total experimental time was estimated by sub-sample number × analytical time per sub-sample) + media preparation time + incubation time. Sub-sample number was calculated as CFU: 72 × 3 × 3 = 648 (72 soil samples × at least 3 dilution factor count × triplicate), Spotting: (72 × 6 × 3) / 18 = 72 (72 samples × each 6 dilution factor and bacterial count × triplicate / 18 sample per agar plate), MPN: (72 × 3) / 3 + 72 (72 samples × triplicate / 3 sample per 9-well plate), FCM: 72 × 3 × 4 = 216 (72 samples × triplicate × standards), EM: 72 × 3 = 216 (72 samples × triplicate), DNA 72 × 3 = 216 (72 samples × triplicate). In the information that can be obtained by each method, ‘Live bacteria (aerobic)’ implies that ‘it could count only live aerobic bacteria’, ‘Live bacteria (aerobic+)’ implies that ‘it could count live aerobic bacteria, dormant bacteria, and VBNC’, ‘Live bacteria (aerobic + anaerobic) + dead + soil’ implies that ‘it could separately count live aerobic and anaerobic bacteria, dead bacteria, and soil particles’, and ‘Bacteria (whole aerobic and anaerobic)’ implies that ‘it could count whole aerobic and anaerobic bacteria without distinguishing between live and dead bacteria.’
(DOCX)
