# Supplementary material for: Revisiting soil bacterial counting methods: Optimal soil storage and pretreatment methods and comparison of culture-dependent and -independent methods
Source: PLoS One. 2021 Feb 10;16(2):e0246142. doi: 10.1371/journal.pone.0246142 (PMC7875414; doi:10.1371/journal.pone.0246142)
Supplement: S1 Fig — (DOCX) [file pone.0246142.s001.docx]

#
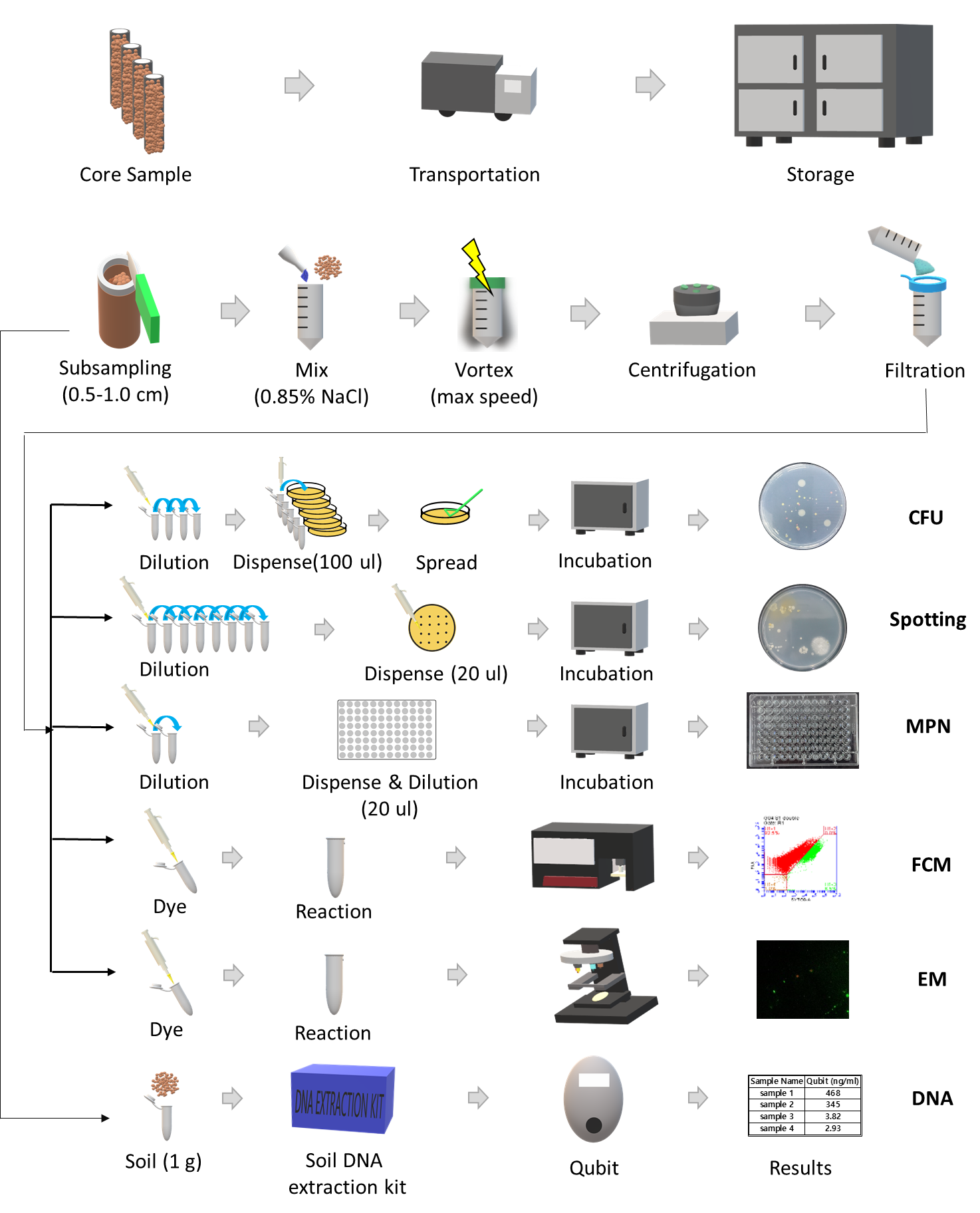


**S1 Fig.** Graphical summary of various pretreatment and counting methods for bacterial numbers in soil samples.
