# Supplementary material for: Revisiting soil bacterial counting methods: Optimal soil storage and pretreatment methods and comparison of culture-dependent and -independent methods
Source: PLoS One. 2021 Feb 10;16(2):e0246142. doi: 10.1371/journal.pone.0246142 (PMC7875414; doi:10.1371/journal.pone.0246142)
Supplement: S2 Fig — Effects of sonication time (a), centrifugation speed (b), and filtration (c) on soil bacterial number during the pretreatment processes. Pretreatment effects were tested by epifluorescence microscope using the garden soil samples collected from around Korea University. These soil samples were stored at 4°C. The pretreatment conditions for sonification time effects (a) included vortexing at maximum speed for 5 min, centrifugation at 1400 × g for 15 min, and filtration through 10 μm filters. The pretreatment conditions for centrifugation speed effects (b) included vortexing at maximum speed for 5 min, sonication at 300 W for 3 min, and no filtration. The pretreatment conditions for filtration effects (c) included vortexing at maximum speed for 5 min, sonication at 300 W for 3 min, centrifugation at 1400 × g for 15 min, with or without filtration through 10 μm filters. Experiments were conducted in triplicate. (DOCX) [file pone.0246142.s002.docx]

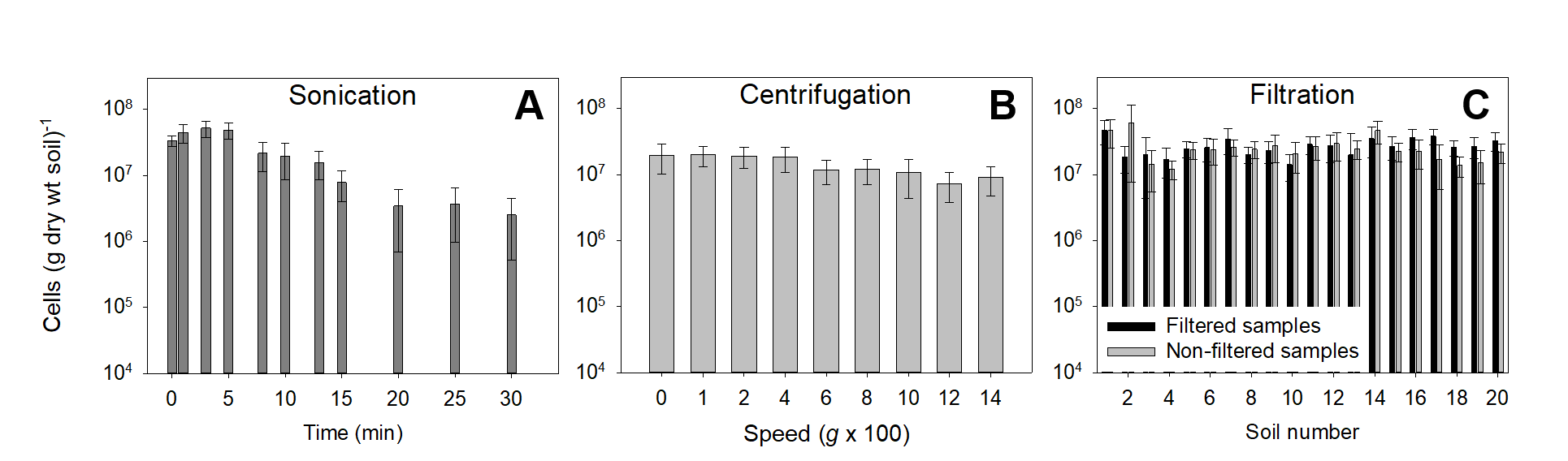


**S2 Fig.** Effects of sonication time (a), centrifugation speed (b), and filtration (c) on soil bacterial number during the pretreatment processes. Pretreatment effects were tested by epifluorescence microscope using the garden soil samples collected from around Korea University. These soil samples were stored at 4 ℃. The pretreatment conditions for sonification time effects (a) included vortexing at maximum speed for 5 min, centrifugation at 1400 *g* for 15 min, and filtration through 10 μm filters. The pretreatment conditions for centrifugation speed effects (b) included vortexing at maximum speed for 5 min, sonication at 300 W for 1 min, and no filtration. The pretreatment conditions for filtration effects (c) included vortexing at maximum speed for 5 min, sonication at 300 W for 1 min, centrifugation at 1400 *g* for 15 min, with or without filtration through 10 μm filters. Experiments were conducted in triplicate.
