# Supplementary material for: Revisiting soil bacterial counting methods: Optimal soil storage and pretreatment methods and comparison of culture-dependent and -independent methods
Source: PLoS One. 2021 Feb 10;16(2):e0246142. doi: 10.1371/journal.pone.0246142 (PMC7875414; doi:10.1371/journal.pone.0246142)
Supplement: S3 Fig — Bacterial cells were counted by epifluorescence microscope using the garden soil samples collected from around Korea University. These soil samples were stored at 4°C. The pretreatment conditions for centrifugation speed effects included vortexing at maximum speed for 5 min, sonication at 300W for 3 min, and no filtration. Experiments were conducted in triplicate. (DOCX) [file pone.0246142.s003.docx]

**S3 Fig.** Effects of centrifugation speed on soil bacterial number during the pretreatment processes. Bacterial cells were counted by epifluorescence microscope using the garden soil samples collected from around Korea University. These soil samples were stored at 4 ℃. The pretreatment conditions for centrifugation speed effects included vortexing at maximum speed for 5 min, sonication at 300W for 3 min, and no filtration. Experiments were conducted in triplicate.
