# Supplementary material for: Revisiting soil bacterial counting methods: Optimal soil storage and pretreatment methods and comparison of culture-dependent and -independent methods
Source: PLoS One. 2021 Feb 10;16(2):e0246142. doi: 10.1371/journal.pone.0246142 (PMC7875414; doi:10.1371/journal.pone.0246142)
Supplement: S4 Fig — The rates were calculated by [(Standard deviation /Average)×100]. Experiments were conducted in triplicate. (DOCX) [file pone.0246142.s004.docx]

**S4 Fig.** Variability rates (%) of microbial cell numbers determined by MPN, spotting and CFU at each soil depth in the farmland. The rates were calculated by $[(Standard deviation$/Average)$\times$100]. Experiments were conducted in triplicate.
