# Supplementary material for: Revisiting soil bacterial counting methods: Optimal soil storage and pretreatment methods and comparison of culture-dependent and -independent methods
Source: PLoS One. 2021 Feb 10;16(2):e0246142. doi: 10.1371/journal.pone.0246142 (PMC7875414; doi:10.1371/journal.pone.0246142)
Supplement: S5 Fig — Each row displayed a different soil. A) No staining; B) SYTO9 staining; C) PI staining; and D) both SYTO9 and PI staining. Experiments were conducted in single. (DOCX) [file pone.0246142.s005.docx]

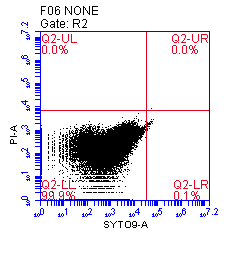

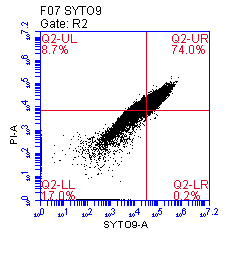

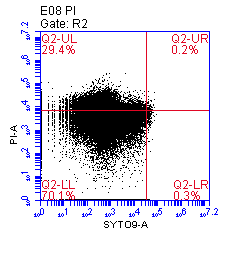

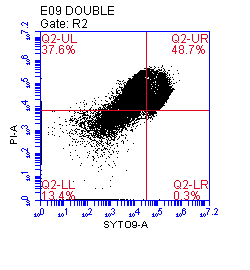

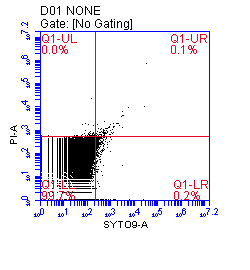

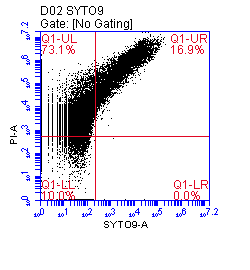

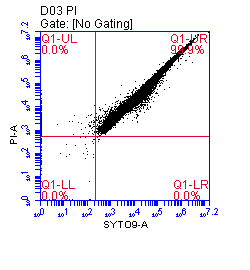

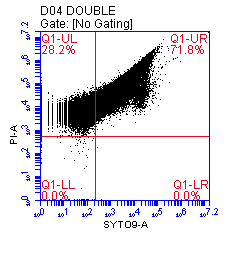

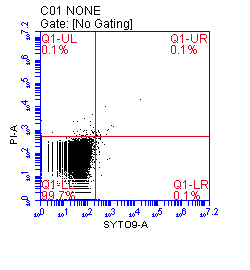

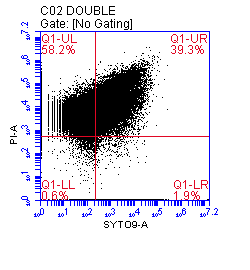

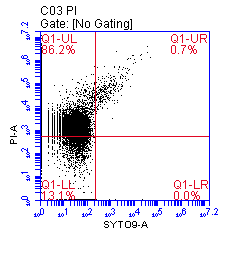

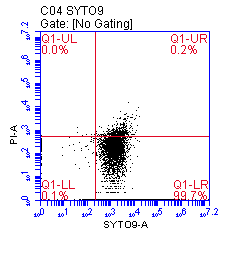


**A**

**B**

**C**

**D**

**S5 Fig.** Scatter graphs were obtained by FCM with three farmland soil samples. Each row displayed a different soil. A) No staining; B) SYTO9 staining; C) PI staining; and D) both SYTO9 and PI staining. Experiments were conducted in single.
