# Supplementary material for: Revisiting soil bacterial counting methods: Optimal soil storage and pretreatment methods and comparison of culture-dependent and -independent methods
Source: PLoS One. 2021 Feb 10;16(2):e0246142. doi: 10.1371/journal.pone.0246142 (PMC7875414; doi:10.1371/journal.pone.0246142)
Supplement: S6 Fig — The samples were stored at 4°C and pretreated by vortexing at maxim speed for 5 min, sonication at 300 W for 3 min, and centrifugation at 1400 × g for 15 min. Experiments were conducted in triplicate. (DOCX) [file pone.0246142.s006.docx]

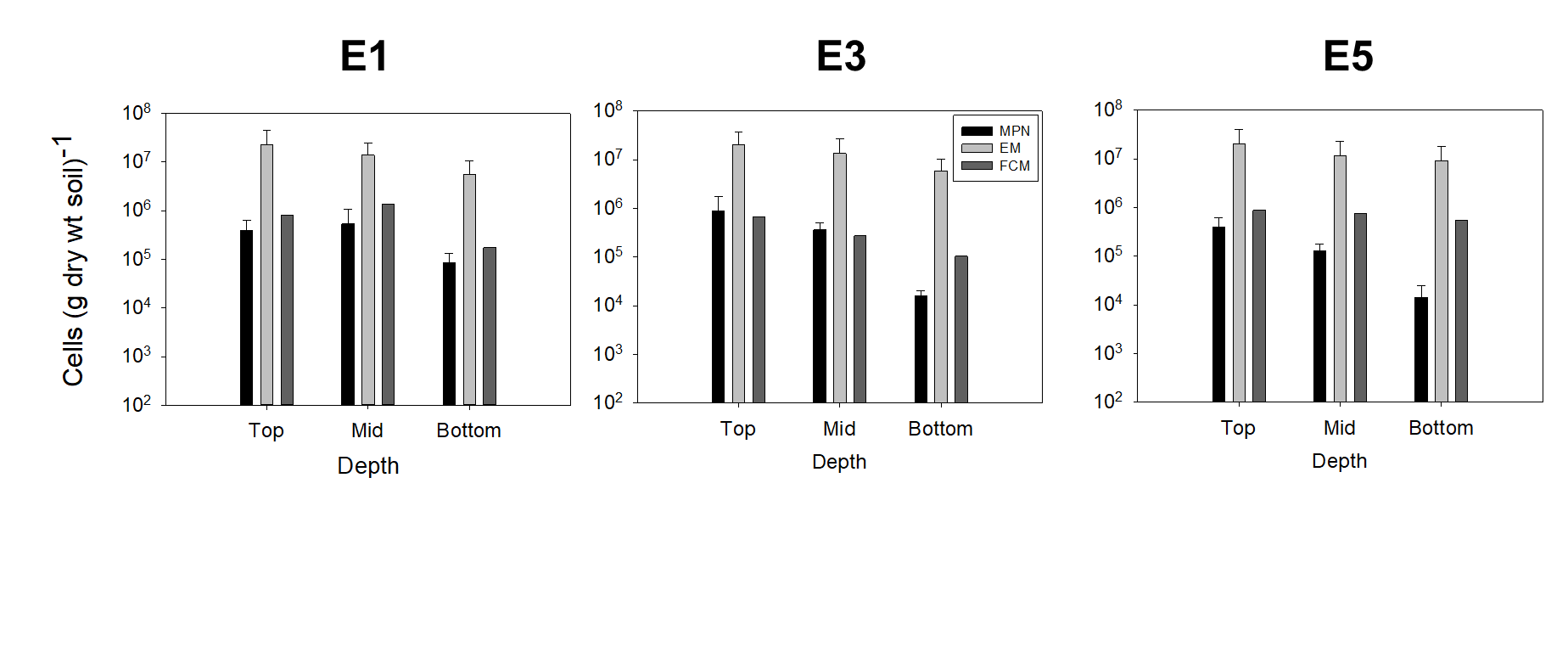


**S6 Fig.** Comparison of live bacterial cell numbers in farmland soil samples determined by MPN, EM, and FCM. The samples were stored at 4 ℃ and pretreated by vortexing at maxim speed for 5 min, sonication at 300 W for 3 min, and centrifugation at 1400 *g* for 15 min. Experiments were conducted in triplicate.
