# Supplementary material for: Revisiting soil bacterial counting methods: Optimal soil storage and pretreatment methods and comparison of culture-dependent and -independent methods
Source: PLoS One. 2021 Feb 10;16(2):e0246142. doi: 10.1371/journal.pone.0246142 (PMC7875414; doi:10.1371/journal.pone.0246142)
Supplement: S1 Table — Comparison of each method was based on a total of 72 soil samples from the farmland. Total experimental time was estimated by sub-sample number × analytical time per sub-sample) + media preparation time + incubation time. Sub-sample number was calculated as CFU: 72 × 3 × 3 = 648 (72 soil samples × at least 3 dilution factor count × triplicate), Spotting: (72 × 6 × 3) / 18 = 72 (72 samples × each 6 dilution factor and bacterial count × triplicate / 18 sample per agar plate), MPN: (72 × 3) / 3 + 72 (72 samples × triplicate / 3 sample per 9-well plate), FCM: 72 × 3 × 4 = 216 (72 samples × triplicate × standards), EM: 72 × 3 = 216 (72 samples × triplicate), DNA 72 × 3 = 216 (72 samples × triplicate). In the information that can be obtained by each method, ‘Live bacteria (aerobic)’ implies that ‘it could count only live aerobic bacteria’, ‘Live bacteria (aerobic+)’ implies that ‘it could count live aerobic bacteria, dormant bacteria, and VBNC’, ‘Live bacteria (aerobic + anaerobic) + dead + soil’ implies that ‘it could separately count live aerobic and anaerobic bacteria, dead bacteria, and soil particles’, and ‘Bacteria (whole aerobic and anaerobic)’ implies that ‘it could count whole aerobic and anaerobic bacteria without distinguishing between live and dead bacteria.’ (DOCX) [file pone.0246142.s007.docx]

**S1 Table.** Summary of cost, experimental time, difficulty, and labor intensiveness of each method and potential information obtained from each method. Comparison of each method was based on a total of 72 soil samples from the farmland. Total experimental time was estimated by sub-sample number × analytical time per sub-sample) + media preparation time + incubation time. Sub-sample number was calculated as CFU: 72 × 3 × 3 = 648 (72 soil samples × at least 3 dilution factor count × triplicate), Spotting: (72 × 6 × 3) / 18 = 72 (72 samples × each 6 dilution factor and bacterial count × triplicate / 18 sample per agar plate), MPN: (72 × 3) / 3 + 72 (72 samples × triplicate / 3 sample per 9-well plate), FCM: 72 × 3 × 4 = 216 (72 samples × triplicate × standards), EM: 72 × 3 = 216 (72 samples × triplicate), DNA 72 × 3 = 216 (72 samples × triplicate). In the information that can be obtained by each method, ‘Live bacteria (aerobic)’ implies that ‘it could count only live aerobic bacteria’, ‘Live bacteria (aerobic+)’ implies that ‘it could count live aerobic bacteria, dormant bacteria, and VBNC’, ‘Live bacteria (aerobic + anaerobic) + dead + soil’ implies that ‘it could separately count live aerobic and anaerobic bacteria, dead bacteria, and soil particles’, and ‘Bacteria (whole aerobic and anaerobic)’ implies that ‘it could count whole aerobic and anaerobic bacteria without distinguishing between live and dead bacteria.’

| Method | | CFU | Spotting | MPN | FCM | EM | DNA |
| --- | --- | --- | --- | --- | --- | --- | --- |
| Sub-sample number | | 648 | 72 | 72 | 864 | 216 | 216 |
| Time (h) | Media preparation | 7.5 | 4 | 3 | 0 | 0 | 0 |
|  | Analysis (sample) | 0.13 | 0.11 | 0.09 | 0.08 | 0.18 | 0.28 |
|  | Incubation | 168 | 168 | 168 | 0 | 0 | 0 |
|  | Total | 259.7 | 179.9 | 177.5 | 69.1 | 38.9 | 60.5 |
| Cost (USD) | Plate | 0.11 | 0.11 | 0 | 0 | 0 | 0 |
|  | Spreader | 0.81 | 0 | 0 | 0 | 0 | 0 |
|  | R2A | 0.02 | 0.02 | 0 | 0 | 0 | 0 |
|  | R2B | 0 | 0 | 0.01 | 0 | 0 | 0 |
|  | 96-well plate | 0 | 0 | 1.69 | 0 | 0 | 0 |
|  | Dye | 0 | 0 | 0 | 5.48 | 5.48 | 0 |
|  | DNA extraction kit | 0 | 0 | 0 | 0 | 0 | 7.22 |
|  | Instrument fee + pretreatment | 0 | 0 | 0 | 17.14 | 4.93 | 1.11 |
|  | Total (quantity × cost) | 703.1 | 9.4 | 122.4 | 19,543.7 | 2,248.6 | 1,731.1 |
| Difficulty (1–10) | | 1 | 3 | 2 | 8 | 6 | 5 |
| Labor-intensiveness (1–10) | | 8 | 6 | 5 | 3 | 9 | 3 |
| Information | | Live bacteria (aerobic) | Live bacteria (aerobic) | Live bacteria (aerobic+) | Live bacteria (aerobic + anaerobic) + dead + soil | Live bacteria (aerobic + anaerobic) + dead + soil | Bacteria (whole aerobic and anaerobic) |
